# Identification of citrus greening based on visual symptoms: A grower's diagnostic toolkit

**DOI:** 10.1016/j.heliyon.2021.e08387

**Published:** 2021-11-13

**Authors:** Mohammad Monirul Hasan Tipu, Md. Mostafa Masud, Raunak Jahan, Artho Baroi, A.K.M.A. Hoque

**Affiliations:** a Plant Pathology Division, Bangladesh Agricultural Research Institute (BARI), Joydebpur, Gazipur, 1701, Bangladesh; b Department of Plant Pathology, Bangladesh Agricultural University, Mymensingh, 2202, Bangladesh; c Department of Aquaculture, Bangladesh Agricultural University (BAU), Mymensingh, 2202, Bangladesh; d Faculty of Agriculture, Bangladesh Agricultural University, Mymensingh, 2202, Bangladesh; e Horticulture Research Centre, Bangladesh Agricultural Research Institute (BARI), Joydebpur, Gazipur, 1701, Bangladesh

**Keywords:** Huanglongbing, *Candidatus liberibacter*, Asian citrus psyllid, Blotchy mottle, Visual symptoms

## Abstract

Citrus greening is one of the world's most serious diseases of citrus that affects all cultivars and causes the systematic death of trees worldwide. The disease is caused mostly by the bacteria *Candidatus Liberibacter* species. The wind, rain, and touch of infected workers cannot transmit this infectious disease. The Asian Citrus Psyllid (ACP), a minuscule insect, is one of the main vectors that transmit the disease by feeding on citrus leaves. Citrus greening management is also quite expensive since no successful treatment for the cure has been found, except to remove all affected vegetables from healthy crops to eliminate the bacterial spread. Citrus greening is also the most challenging task, as signs of other citrus diseases and nutritional deficiencies are identical. The major signs of this disease are asymmetrical, blotchy mottling patterns on leaves. Here, several visible indications of citrus greening were addressed, which will enable farmers at the root level to detect and avoid this condition prior to its having a dramatic influence on citrus plantations. We also talked about the pen test method to determine symptoms as symmetrical or asymmetrical throughout the mid-vine, regardless of whether they are impacted by citrus greening or lack of nutrients.

## 1 Introduction

Citrus greening (huanglongbing; HLB) is one of the deadliest citrus diseases and affects the production of citrus wherever it exists. Statistics suggest that in more than 58 nations, the tropics and subtropics of Africa and Asia, including India and Bangladesh, have been reported (Spann et al., 2008; Tipu et al., 2017; Braswell et al., 2020; Vashisth and Kadyampakeni, 2020). The disease was surprisingly called "Vein Phloem Degeneration" (Tirtawidjaja et al., 1965). Among all the citrus diseases, citrus greening affects all citrus fruits (Bové, 2006; Gottwald, 2010; Wang et al., 2017). It is primarily a bacteria-induced disease that significantly reduces yield and exacerbates the economic value of citrus fruits, ultimately dispatching the entire tree (Bové and Garnier, 2002; FAO, 2012; NASEM, 2018; Tipu et al., 2020). This disease is caused by the phloem limited, gram-negative *Candidatus Liberibacter* (α-Proteobacteria) bacteria, of which three (3) species are africanus, americanus, and asiaticus (Bové, 2006; Wang and Trivedi 2013; Li et al., 2017; Rao et al., 2018), commonly known as (CLaf) (Jagoueix et al., 1994) (CLam) (Teixeira et al., 2005) and (CLas) (Jagoueix et al., 1994). For species of two highly moving psyllids, *Diaphorina citri* Kuwayama (Asian Citrus Psyllids) for CLas and CLam, and *Trioza erytreae* Del Guercio (African Citrus Triozide) for CLaf, start inoculation during the feeding stage (McClean and Oberholzer, 1965; Capoor et al., 1967; Hall et al., 2013; Qasim et al., 2018).

The main attraction for CLas and Clam species is young growing leaves (Halbert and Manjunath, 2004; Grafton-Cardwell et al., 2013; Hall et al., 2013), but it is obvious that those species exacerbate the vascular symptoms of the plant (Li et al., 2006, 2009; Tatineni et al., 2008; Lopes et al., 2009; Coletta-Filho et al., 2014; Johnson et al., 2014). One study (Li et al., 2006) reported that the largest bacterial titers were recorded in the petioles and mid-ribs of the leaf, and the same bacterial populations were also observed in the peduncle, columella, and leaf midribs compared to seeds, young shoots, flower buds, flowers, and bark, which eventually opened the door to diagnosing foliar tissue that was used as a common approach in previous approaches. One of the interesting facts about the Asian citrus psyllid (ACP) is that it can carry the bacterium for many days, and when feeding on an uninfected plant, it has the ability to transfer the bacterium. It takes just 30 min, however, to suck the bacteria from the infected leaves (FAO, 2013; Molki et al., 2019). Recently, a few results showed that the host's root system was first destroyed by that bacterium as well as from the feeding location; it went through a process of multiplication in which it increased its population and then began its journey to leaves (Johnson et al., 2014; Rao et al., 2018). *Liberibacter* is one of the other bacteria (*Liberibacter*, *Serratia marcescens* (Zhang et al., 2005); *Candidatus Phytoplasma*, *Spiroplasma*, and *Candidatus Phlomobacter fragariae* (Zreik et al., 1998)) that could easily live within plant cells, mainly within phloem sieve cells.

Due to the movement of bacteria inside the roots and leaves, the phloem tissues of these two areas are radically blocked. Thus, hindrance to the movement of nutrients and sugars in the internal tissues (Bendix and Lewis, 2018), results in loss of leaves, uneven fruit size, which can affect the taste and texture of fruits, as well as premature fruit drop and eventually the death of the tree (FAO, 2013). It could also be disseminated during the grafting process if the grafting tools have been taken from infected plant tissue (Grafton-Cardwell et al., 2006; Garcés-Giraldo, 2012; León and Kondo, 2017). It cannot be spread by wind, rain, or contact with contaminated personnel, unlike other infectious diseases. In the citrus growing areas of the world, its insect vectors have been most observed, and this may occur due to gigantic movements of plants and insects around the globe (Tolba and Soliman, 2015) as well as some calamities such as hurricanes or storms that have played an important role in expanding the Asian citrus psyllids over vast distances (Gottwald 2010; Wang et al., 2017). Among the three *Candidatus Liberibacter* species, there is the fact that their ecological adaptation is not the same to each other considering temperature. If we represent CLas, which fosters when the temperature is over 30 °C, which manifests as a heat-tolerant species (Hall et al., 2013), and the CLaf species, which adaptation is closely associated when the temperature is below 30 °C. That's why in the case of altitudinal distribution of the two pathogen species, ecological preferences are first (Jagoueix et al., 1994).

A valid reason for losing the commercial value of fruits affected by HLB is that, along with the yield reduction, falling market price, and management of disease and production is very costly, which severely affects the economic condition. Also, citrus greening deteriorates the marketability of fruits in the agricultural field (Inoue et al., 2020). When a tree begins to be infected, it is totally a loss project to apply chemical pesticides because they cannot be cured completely with pesticides. In addition, young plants are more susceptible to this disease, and it is therefore a very laborious job for farmers to replace the young plants that have been affected so far. Recently, no fruitful method has been developed to cure this deadliest disease, except to remove all infected plants from good ones to eliminate the spread of the pathogen (Inoue et al., 2020). One of the most difficult tasks is to identify citrus greening and other disorders due to the likeliness of HLB and other citrus diseases such as Citrus tristeza virus and problems with nutrient deficiency, which are often confused (McClean and Schwarz, 1970; Albrecht et al., 2016).

It is difficult to conclude a correct diagnosis at the onset of the disease. In comparison with warmer months, HLB symptoms in cooler months are more apparent, McCollum and Baldwin (2016) noted. One interesting fact is that for several months or years without manifesting any symptoms (a symptomless phase), HLB-infected plants can bear huge amounts of bacteria, which is an important factor for infecting other uninfected plants. Although symptoms can be found throughout the year, but most likely from September to March, there has been a highly accelerated symptomatic visibility of that disease. Symptoms are easily seen in the shade or overcast days, as well as in almost all parts of the plants, including the canopy, leaves, twigs, and fruit where infection occurs; the entire tree decreases rapidly as the disease progresses.

## 2 Leaf symptoms

**Figure 1:**
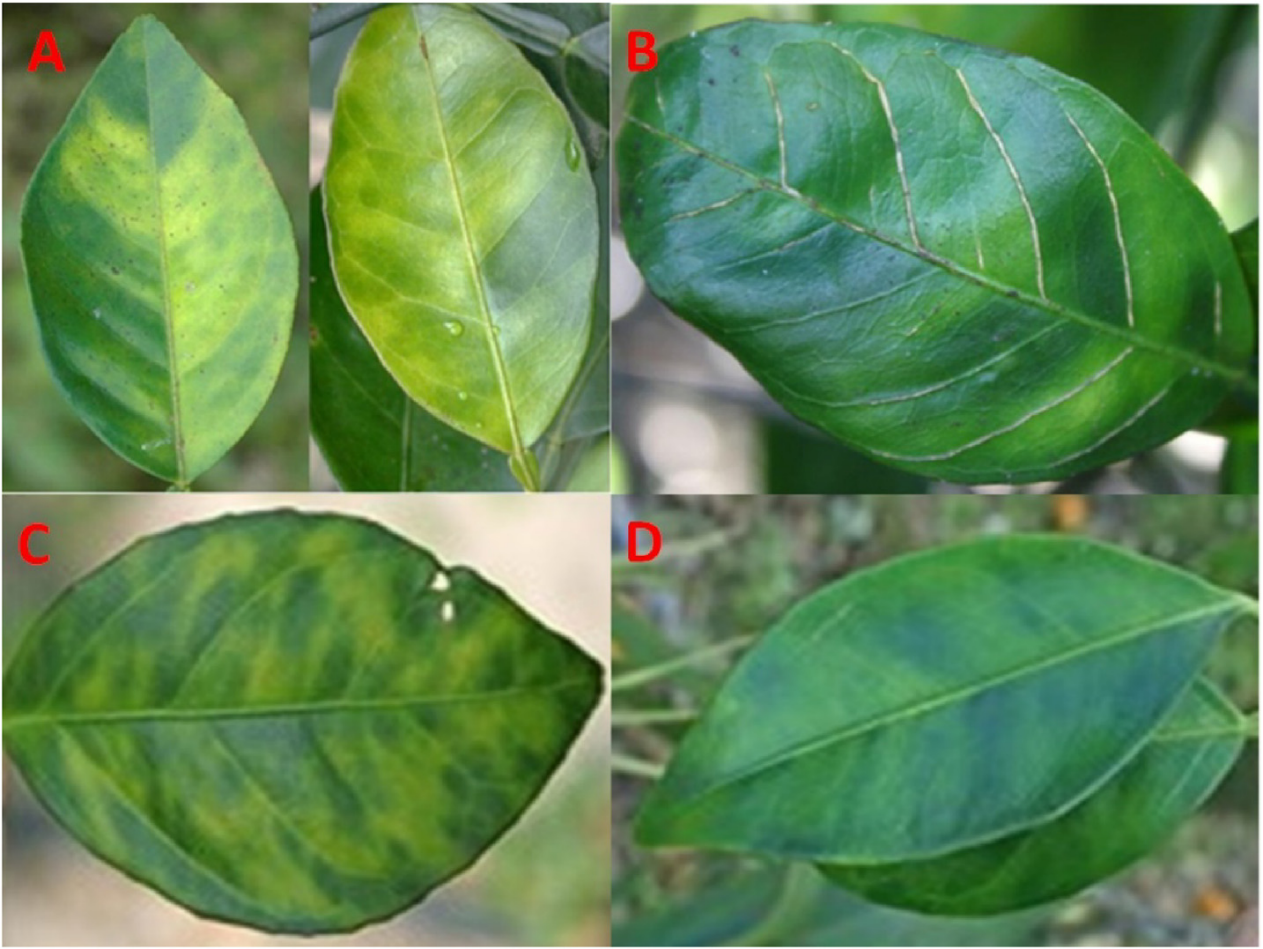
Diagnostic blotchy mottle leaf pattern of HLB (A), vein corking (B), green islands (C), and blotchy mottle (D) in leaves.

Blotchy mottling patterns on the leaves are asymmetrical using the mid-rib as a central line, which is by far the inevitable diagnostic greening symptom, and it is between said as severe infection and incidentally similar to the sign of Fe deficiency when leaves turn discolored yellow (Inoue et al., 2020) (Figure 1). Nevertheless, the symmetrical pattern would be observed if plants suffered from nutrients and other disorders (i.e., almost exact replicas of each half using the mid-rib as a mirror). One study (Ammar et al., 2013) found that a huge number of nymphs were found on both sides of the leaf, approximately 64.50 percent and 35.50% on the lower (abaxial) side of the leaf and the upper (adaxial) side of the leaf, respectively, where more numbers were found on the lower side of the leaf than on the upper leaf. Usually, plants show these nutrient deficiencies and blotchy mottle affected by HLB. However, these symptoms are not definitive HLB symptoms. The blotchy mottle is mainly present in young or mature leaves anywhere in the tree canopy. For greening, vein corking and yellow veins alone are not diagnostic. If present, however, check the tree canopy for blotchy mottle symptoms as well: mid-vein, "corky" mid-veins/under-rib, interveinal chlorosis, bright yellow shoots among a green canopy, pronounced/prominent.

## 3 Fruit symptoms

**Figure 2:**
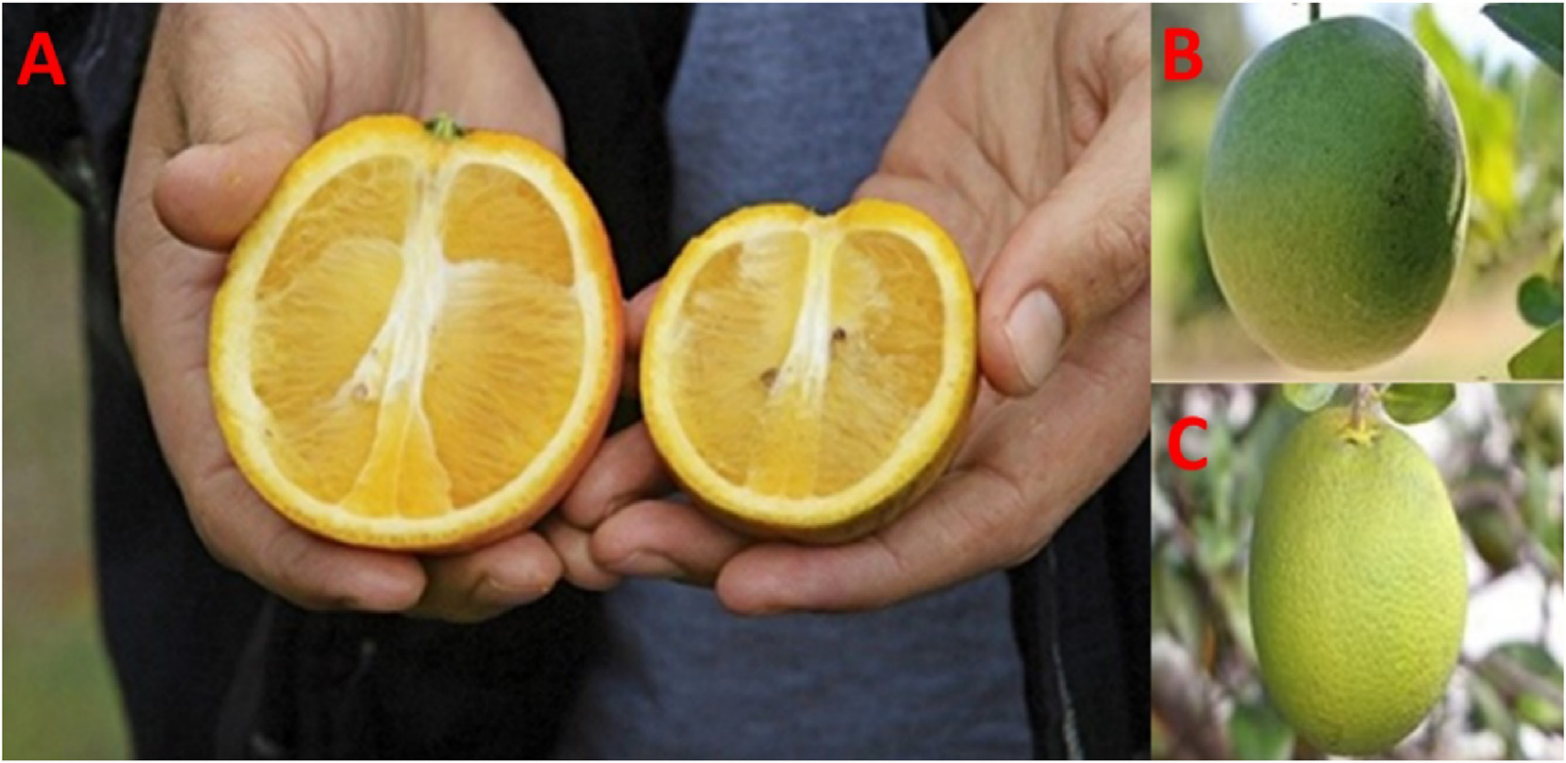
Huanglongbing infected orange trees bear small and lopsided fruits (right) relative to healthy fruits (left) (A) (Credit: Tom Benitez), Lopsided fruit (B), and Misshapen fruit (C).

As an infected tree deteriorates rapidly, the fruit may also begin to display signs of disease. The color of the stylus end shows the yellow/orange color when healthy fruits reach maturity, and the green color is associated with the peduncle end. While fruits are affected by HLB disease, on the other hand, the peduncular end is yellow-orange and the stylus end is still green (Bové, 2014) (Figure 2). The fruit can be lopsided, oblong, distorted, or small and green. Inversion of fruit color formation (fruit yellowing from top to bottom on citrus varieties of orange color). A small number of sour oranges are tolerant among all varieties, and their symptoms are not as dreadful as compared to others. Fruit dropping starts in its early stage of life due to having symptomatic fruits in infected trees, which means immature fruits are falling off and sub-sequencing (Dala-Paula et al., 2019). Although many of these symptoms can be confused with deficiencies and disorders, the symptoms usually manifest in one branch or part of the tree at first, then slowly spread throughout the entire canopy, while deficiencies and disorders within the canopy and among trees are usually more uniform.

## 4 Whole tree symptoms

**Figure 3:**
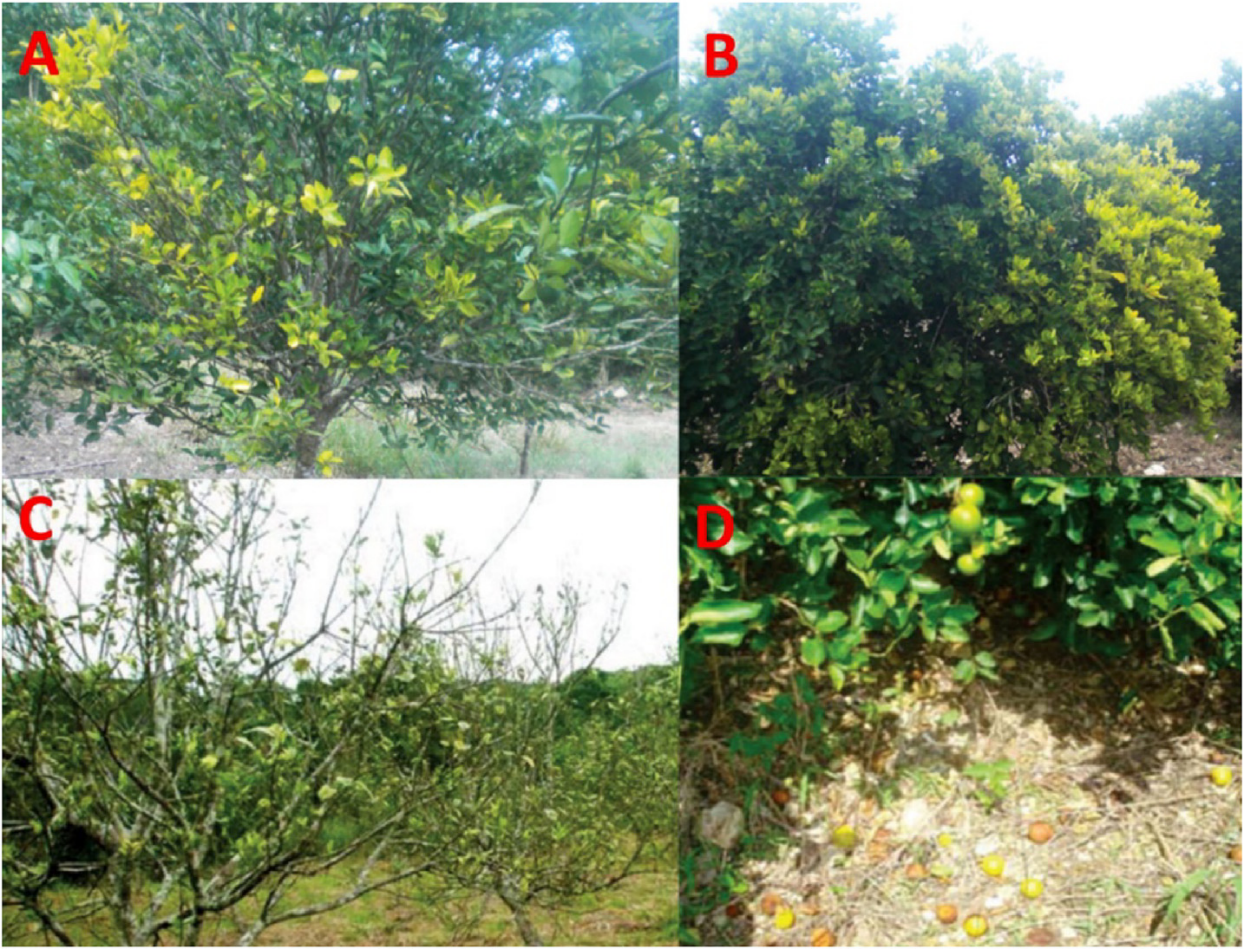
An HLB-affected tree in which the symptomatic leaves (A,B), shoot dieback (C), and Excessive fruit drop (D).

In narrow upright leaves, as well as in yellow shoots, the blotch mottle and yellow veins are seen (Figure 3). In addition, several symptoms, including shoot dieback, stunting, off-season bloom, overall yellow appearance, and a drop in fruit, have been recorded. Symptoms were only seen on one part of the canopy now and then. It has been a common incident that the root system is particularly destroyed by CLas infected trees. Thus, when associated with HLB disease, a large number of root losses occur (Graham et al., 2013). Before the really visible symptoms appear, the initiation of root dieback is observed. The proportion of root-shoot ratio decreases over time in HLB affected trees (Johnson et al., 2014). One thing is clarified by (Shahzad et al. (2020) that healthy trees have a higher amount of biomass for roots, shoots, and lead compared to the HLB affected trees, and the data revealed remarkably that when affected by HLB, nearly 40 %–50 % less root biomass is recorded compared to healthy trees.

## 5 Differences between HLB and other nutrient deficiency problems

Diagnosing the mineral nutrient deficiency from greening symptoms is crucial because it is also responsible for leaf yellowing due to the absence of a few nutrients like zinc, iron, manganese, and calcium resulting from the mineral deficiencies, which are also similar to greening symptoms. Diffuse asymmetric mottle is produced when HLB affects the plant, which can be shown as atypical patches of light green or yellow in contrast to the normal green color of the leaf. It has been difficult to distinguish between HLB symptoms and nutrient deficiency symptoms, but a cautious and precise identification could isolate them. At the later stages of the disease, nutrient deficiency symptoms are often exposed, and for each symptom, it will have different patterns, but one thing is certain: patterns always occur across the mid-vein.

**Figure 4:**
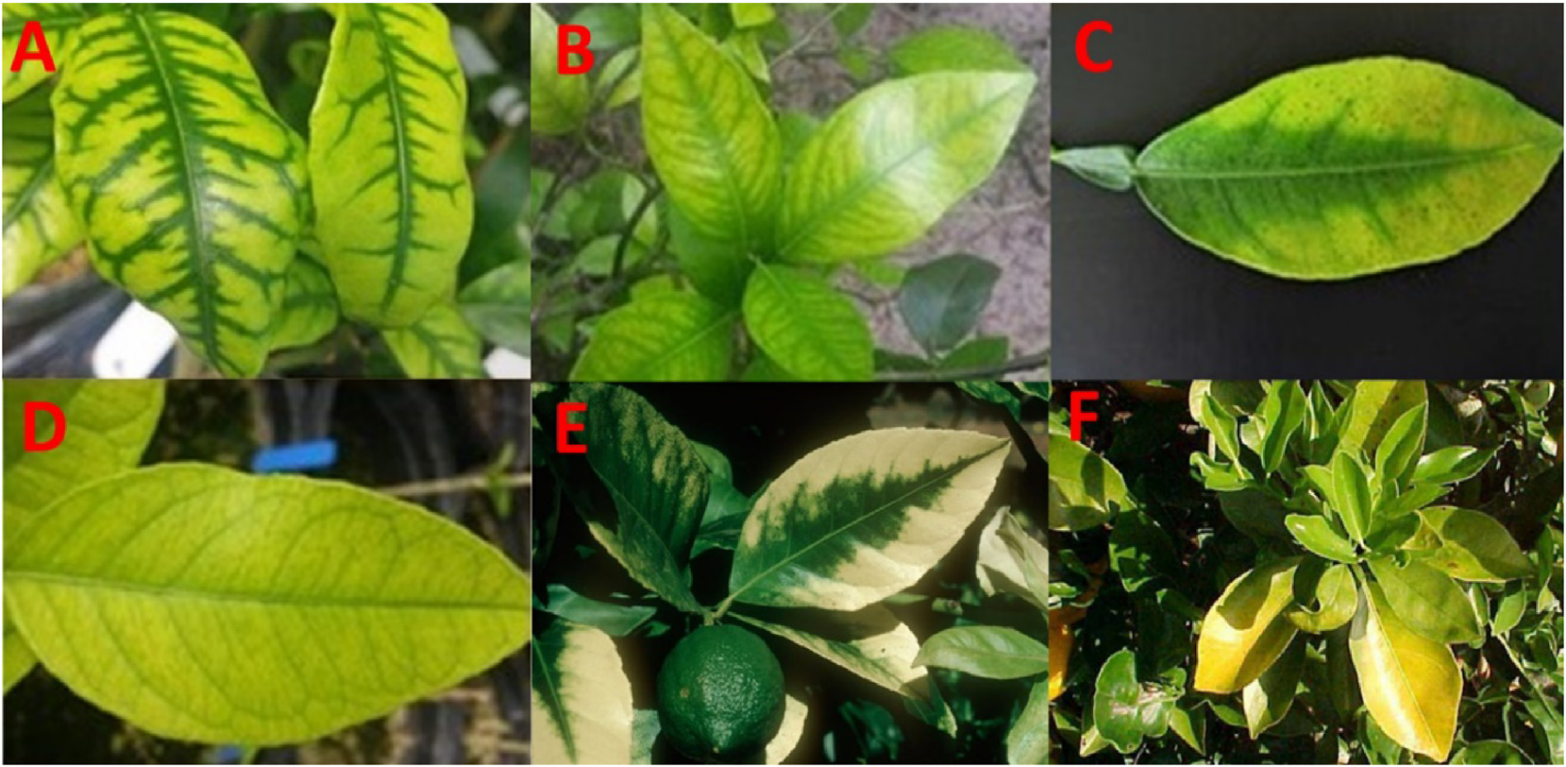
Different nutrient deficiency symptoms, e.g. Zinc deficiency (A), Manganese (Mn) deficiency (B), Magnesium deficiency (C), Iron deficiency (D), Calcium deficiency (E), and Nitrogen deficiency (F).

Zinc (Zn) deficiency: In each leaf, an interveinal yellow mottle is observed, which is symmetrical to the central vein, but at the time of extreme deficiency, the leaves are often small, narrow, and chlorotic; other than that, HLB could also occur (Figure 4A).

Manganese (Mn) deficiency: It is almost similar to zinc deficiency, but the combination of colors, which is between light green and dark green, is a bit different; young leaves, however, have this kind of deficiency (Figure 4B).

Magnesium (Mg) deficiency: In the event of this deficiency, an upturned "V" form was manifested, with green patches of color on both sides of the central vein differentiating from the yellow of the remaining leaves, and aged leaves appeared to be more affected than others (Figure 4C).

Iron (Fe) deficiency: Normally, the leaves with light green and young, as well as almost pale, are affected by iron deficiency (Figure 4D).

Calcium (Ca) deficiency: A deficiency of calcium in citrus is expressed as a fading of the chlorophyll along the leaf margins and between the main veins during the winter months. Small necrotic (dead) spots can develop in the faded areas. Calcium deficiency produces small, thickened leaves and causes loss of vigor, thinning of foliage and decreased fruit production (Figure 4E).

Nitrogen deficiency: Due to nitrogen deficiency, the whole leaf color turns light yellow, and the size of the leaf is reduced (Figure 4F).

## 6 How to detect greening

It is always the trickiest job to identify greening disease in a grower's orchard, especially when trees face impoverished health. One of the essential symptoms for diagnosing a greening disease is blotchy mottle, and it has been recognized as a typical symptom by which anyone can detect the disease, but numerous studies have revealed that during the months from June to August (summer months), it is quite tough to pinpoint the disease, as at that time, trees are actively raising their leaves. Therefore, it comes out that one has the best chance of distinguishing this particular disease from September to May (fall, winter, and spring) (Spann et al., 2008). As a result, the branch affected by the diseased leaf must be removed, and it is necessary to inspect the inside of the tree. When the spread of the disease is severe in the tree, it is suggested to check whether there are any symptoms in the fruit or not. It is a great approach to locate the specific infected tree by making a visible spot on the particular branch to sort out healthy and unhealthy trees.

## 7 Identification of symptoms of citrus greening at field level

**Figure 5:**
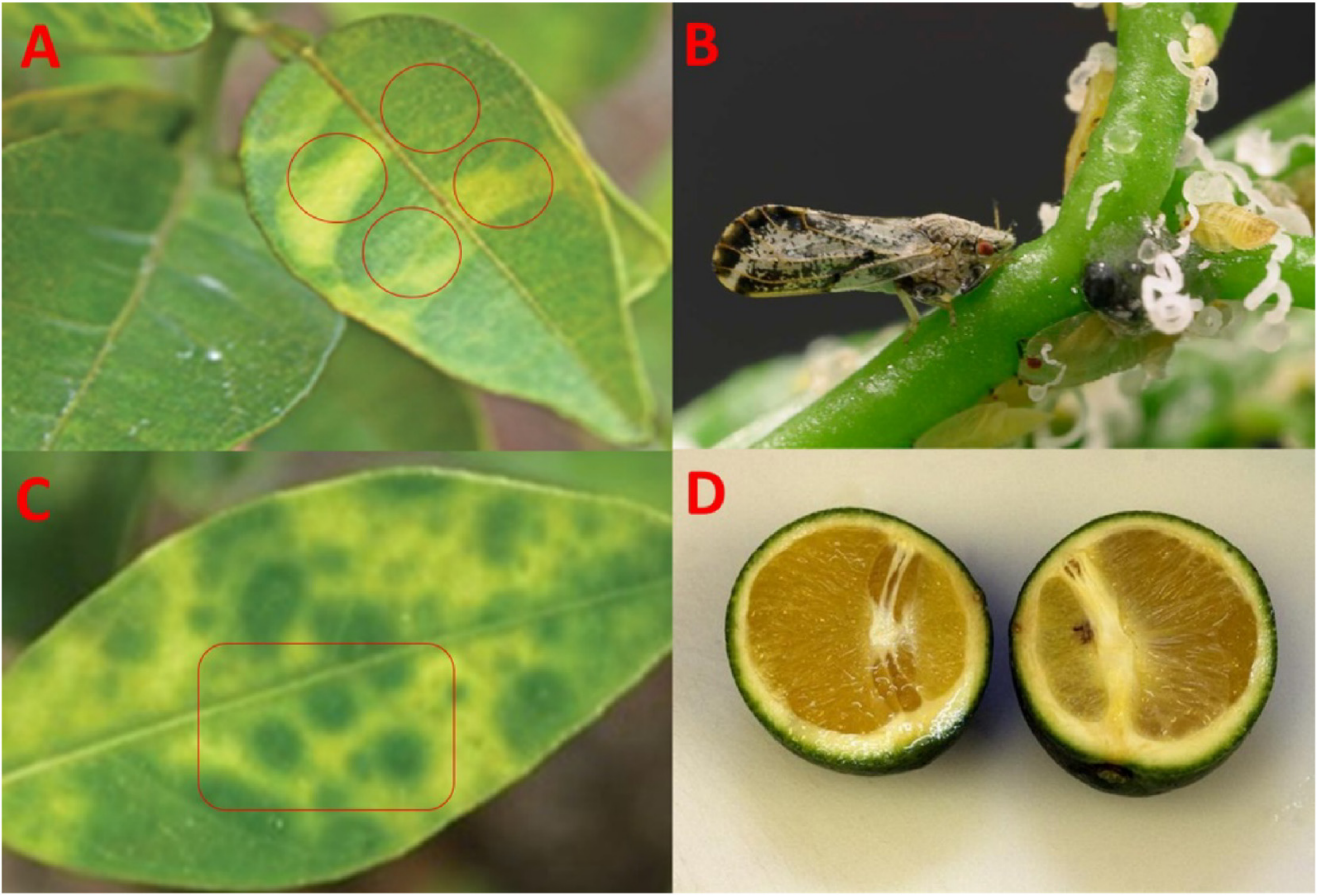
Diagnostic symptoms/signs of huanglongbing at field level showing dissimilar blotchy mottle pattern on both sides (A), presence of citrus psyllid nymph and adult (B), green Island like view on the leaf surface (C), and lopsided fruit with aborted seeds (D).

It has been studied that the expressive symptoms of HLB disease occur in several parts of the trees, including, most importantly, in leaves, fruit, twigs, and branches. However, it is always found to be difficult to detect that disease as it expresses some variable symptoms, which is tough to understand at the grower's level without knowing their patterns precisely, as well as some symptoms may match with other citrus diseases. In this paragraph, we have tried to come up with some important and ambiguous symptoms so that it will immensely assist root level farmers in taking the necessary steps to identify and prevent these diseases before they drastically ruin the whole disease. Here, we focused on some crucial visual symptoms: in Figure 5A, the blotchy mottle present does not resemble both sides of the mid-vein, which is a principal symptom, and leaf notching symptoms were recorded due to intense psyllid feeding. In the case of Figure 5B, psyllid nymphs and adults are seen at the lower portion of leaves and twigs, but the size of nymphs is too tiny. Moreover, now and then the mid vein is radically exposed with yellow color from top to bottom. Apart from that, a standard symptom like a green island on a yellow leaf is manifested in some foliage (Figure 5C). When it comes to fruit, the size of the fruits is lessened, the fruits are misshapen and lopsided fruits (Figure 5D). In addition, the taste is more bitter and salty than the normal one.

## 8 Pen test

**Figure 6:**
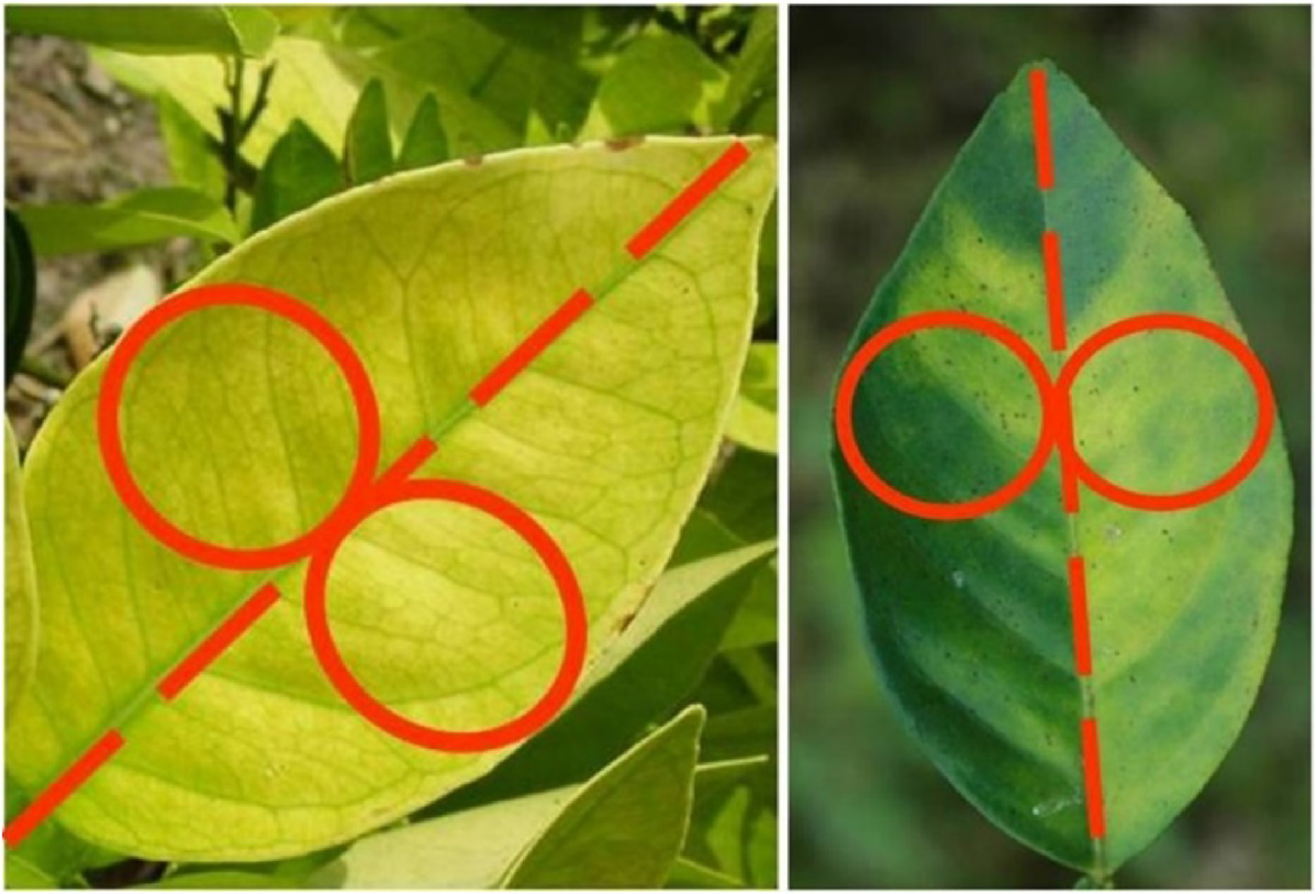
Nutrient deficiency (left) compared to HLB symptoms (right); notice the symmetry on either side of the midvein in the nutrient-deficient leaf compared to the symptoms' asymmetrical pattern HLB affected leaf.

A "pen test" is the most popular means of distinguishing HLB symptoms from nutritional deficiency. This test is well known as a visual diagnosis test, which precisely measures the homogenous patterns on both surfaces of the leaf. With the help of this method of determining the symptoms as symmetrical or asymmetrical across the mid-vein, whether they are HLB-affected or lack nutrients (Vashisth et al., 2016; Vashisth and Kadyampakeni, 2020). To conduct this, we need to mark the leaf by making two circles on antipodal halves, which should be represented at the central vein beside one another as shown in Figure 6. Therefore, with the help of this, it is more convenient to detect greening symptoms or not, such as, if the circles represent the identical symptoms, then they must be symmetrical, which indicates no sign of greening. But naturally, nutrient deficiency symptoms show an exactly alike pattern in both circles, apart from that HLB blotchy mottle pattern doesn't resemble the areas of the two circles; thus, it concludes that it has greening symptoms. The fact that HLB-affected trees also commonly have nutrient deficits must be considered, so the distinction between deficiencies and HLB symptoms should be made and deficiencies rectified by correct fertilizer formulation.

## 9 Conclusion

It is difficult, and sometimes not affordable, for growers to detect citrus greening by using molecular tools. This diagnostic guideline will be an easy toolkit for detecting huanglongbing in citrus orchards with limited technical knowledge. However, this review emphasizes field diagnosis by visual investigation. Additional detection through molecular techniques is necessary to study and confirm the pathogen.

## Declarations

### Author contribution statement

All authors listed have significantly contributed to the development and the writing of this article.

### Funding statement

This research did not receive any specific grant from funding agencies in the public, commercial, or not-for-profit sectors.

### Data availability statement

Data included in article/supplementary material/referenced in article.

### Declaration of interests statement

The authors declare no conflict of interest.

### Additional information

No additional information is available for this paper.
